# AngioJet thrombectomy with extracorporeal membrane oxygenation support for an acute large-scale pulmonary embolism with bilateral atrial thrombosis: a case report of catastrophic antiphospholipid syndrome

**DOI:** 10.3389/fcvm.2024.1409775

**Published:** 2024-07-02

**Authors:** Jianyu Ji, Lei Jiang, Wei Wang, Xinyu Chi, Jinda Dong, Liqiu Lu, Minyan Huang, Xiutian Wei, Guangbao Pang, Jing Pang, Bin Xiong, Shulin Xiang

**Affiliations:** ^1^ Guangxi Academy of Medical Sciences, Nanning, Guangxi, China; ^2^ Department of Intensive Care Unit, The People's Hospital of Guangxi Zhuang Autonomous Region, Nanning, Guangxi, China; ^3^ Research Center of Communicable and Severe Diseases, Guangxi Academy of Medical Sciences, Nanning, Guangxi, China; ^4^ Guangxi Health Commission Key Laboratory of Diagnosis and Treatment of Acute Respiratory Distress Syndrome, Guangxi Academy of Medical Sciences, Nanning, Guangxi, China; ^5^ Department of Blood Transfusion, The People's Hospital of Guangxi Zhuang Autonomous Region, Nanning, Guangxi, China

**Keywords:** catastrophic antiphospholipid syndrome, acute large-scale pulmonary embolism, bilateral atrial thrombosis, AngioJet thrombectomy, extracorporeal membrane oxygenation, case report

## Abstract

**Background:**

Catastrophic Antiphospholipid Syndrome (CAPS), a severe systemic autoimmune disorder, predominantly causes life-threatening multi-organ failure, with a high mortality rate. It primarily affects small vessels, seldom impacting large vessels. Notably, acute massive pulmonary embolism (PE) with bilateral atrial thrombosis is an exceptional occurrence in CAPS. Acute pulmonary embolism (PE) is a common cardiovascular disease that progresses rapidly and has a high mortality rate. Acute massive PE combined with bilateral atrial thrombosis has an even higher mortality rate. PE treatments primarily include pharmaceuticals, catheter interventions, and surgical measures, with integrated treatment strategies demonstrating promising outcomes in clinical practice. Extracorporeal membrane oxygenation (ECMO) can provide cardiopulmonary support for the treatment of high-risk PE patients and is a proven therapeutic measure.

**Methods:**

This report presents the case of a 52-year-old male admitted due to fever and sudden onset of impaired consciousness, with cardiac ultrasound and pulmonary artery CT angiography revealing an acute large-scale pulmonary embolism accompanied by bilateral atrial thrombosis, with the condition rapidly worsening and manifesting severe respiratory and circulatory failure. With ECMO support, the patient underwent a thrombectomy using an AngioJet intervention. The diagnosis of CAPS was confirmed through clinical presentation and laboratory examination, and treatment was adjusted accordingly.

**Results:**

The patient made a successful recovery and was subsequently discharged from the hospital.

**Conclusion:**

In CAPS patients, the rare instance of acute massive PE accompanied by bilateral atrial thrombosis significantly risks severe respiratory and circulatory failure, adversely affecting prognosis. Early initiation of ECMO therapy is crucial, offering a vital opportunity to address the root cause. In this case report the patient was successfully treated with an AngioJet thrombectomy supported by ECMO.

## Introduction

Catastrophic Antiphospholipid Syndrome (CAPS), an autoimmune disorder, is marked by rapid-onset multi-organ thrombosis and functional failure, typically manifesting within a week (1). This syndrome is present in about 49.1% of Antiphospholipid Syndrome (APS) patients and is associated with a high mortality rate, ranging from 30% to 50% (2). The severity of CAPS often necessitates intensive care unit admission due to multiple organ failure, the primary cause of mortality cause (3). Renal complications are evident in 73% of CAPS cases, with pulmonary involvement following at 58.9%. Notably, pulmonary embolism is observed in 24.9% of cases, whereas the co-occurrence of pulmonary embolism with bilateral atrial thrombosis is exceedingly rare. The central nervous system is implicated in 55.9% of cases, and cardiac complications are seen in approximately 49.7% of patients. The grave risk of mortality linked with CAPS highlights its clinical urgency, underscoring the need for prompt diagnosis and aggressive treatment in affected individuals (4).

Acute PE, a common fatal cardiovascular disease, is caused by endogenous or exogenous thrombi blocking the pulmonary artery, leading to pulmonary circulation and right heart dysfunction. The annual incidence of acute PE is 39–115 cases per 100,000 people, and over 300,000 people die from PE each year (5, 6). The occurrence and mortality risks of acute PE are closely associated with age; for people over 40 years, the risk approximately doubles with every additional decade of age. Therefore, as age increases, the incidence and mortality rates of acute PE steadily rise (7). Patients with acute PE can be asymptomatic, and the initial manifestation can be sudden death.

The occurrence of acute massive PE combined with bilateral atrial thrombosis is extremely rare, and the mortality rate is extremely high. The severity of the pulmonary artery occlusion is closely related to its impact on cardiac function and the prognosis of patients with PE, among whom patients with hemodynamic instability or even cardiac arrest have an extremely poor prognosis (8, 9). Clinical therapeutic approaches for PE encompass pharmaceuticals (anticoagulants and thrombolytics), catheter procedures (catheter-directed thrombolysis, catheter embolectomy, and catheter-assisted fragmentation techniques), and surgical intervention (surgical thrombectomy) (10). Research in recent years indicates that ECMO can provide cardiopulmonary support for high-risk PE patients and help improve patient prognosis. Additionally, treating PE through surgical thrombectomy and reperfusion, regardless of when ECMO is implemented, can yield positive results for patient prognosis (11). We report the case of a male patient with acute massive PE combined with bilateral atrial thrombosis who also presented as hemodynamically unstable. The patient was successfully treated with an AngioJet thrombectomy supported by ECMO.

## Case description

**Figure 1:**
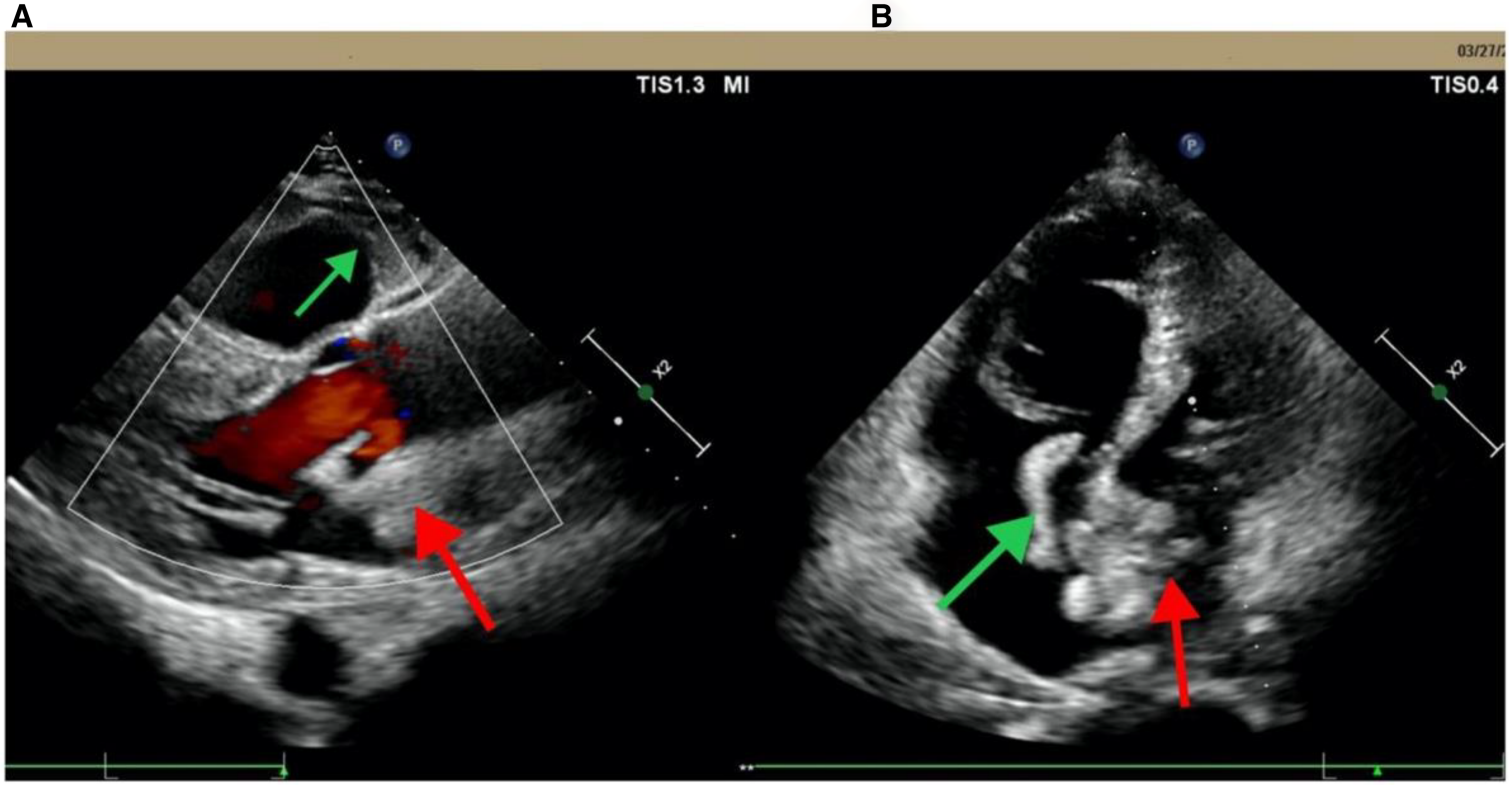
Echocardiogram from Pingguo People's hospital shows biatrial thrombosis. (**A**) Parasternal long-axis section; (**B**) apical four-chamber view. The red arrow points to the left thrombus in the left atrium; the green arrow points to the right atrium thrombus.

A 62-year-old male patient was admitted to the People's Hospital of Guangxi Zhuang Autonomous Region on March 28, 2023 due to fever and sudden syncope. The patient had a history of hypertension and diabetes for over 20 years without regularly taking antihypertensive and hypoglycemic medications. Additionally, he had had depression for more than 10 years and had been on long-term treatment with sertraline and risperidone. The patient had been admitted to a local hospital due to fever and syncope 2 days previously, where echocardiogram findings indicated an enlargement of the right atrium and ventricle and thrombi within both the left and right atria (Figure 1). After admission, the patient was provided with endotracheal intubation and assisted breathing using a ventilator, antishock therapy, vasopressor therapy with vasoactive drugs, circulation improvement, myocardial nutrition, anticoagulation treatment (drug usage and dosage: rivaroxaban 20 mg Qd), antiplatelet aggregation, acid suppression and gastric protection, and other symptomatic supportive treatments to maintain stability. The patient's condition did not improve, and considering the severity of the condition, the local hospital recommended transfer to a higher-level hospital for treatment.

**Figure 2:**
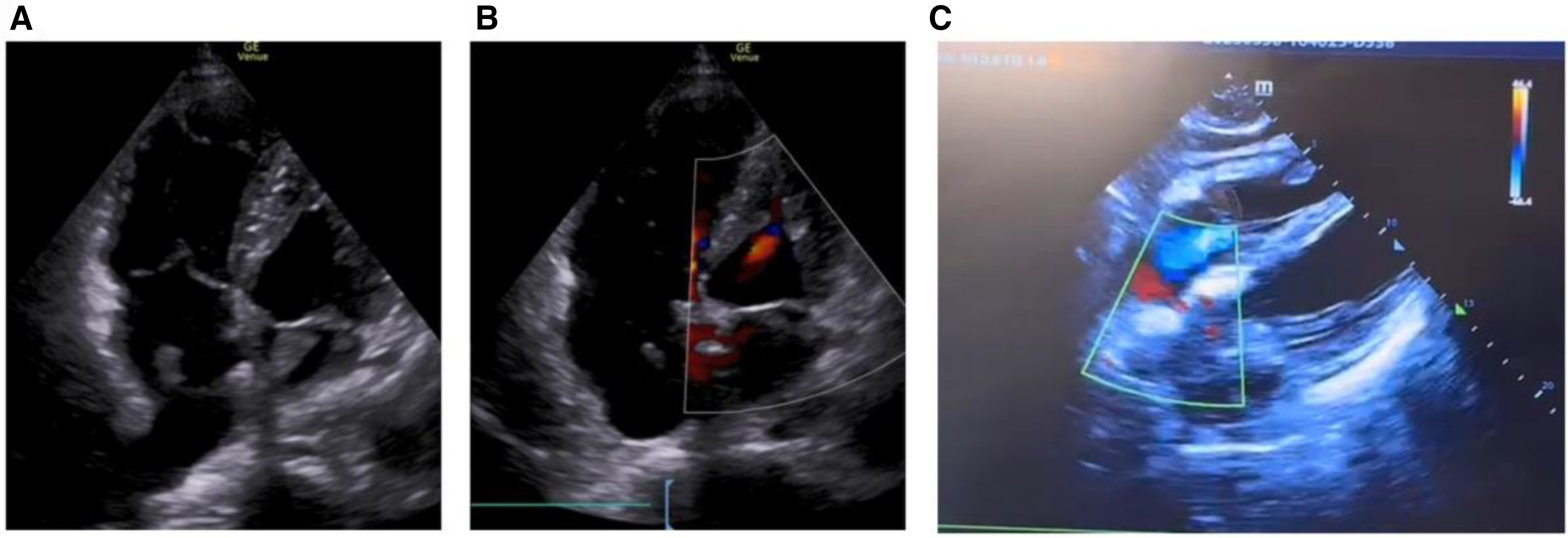
Echocardiogram shows biatrial thrombosis, atrial defect or patent foramen ovale. (**A**) Apical four-chamber view, biatrial thrombus; (**B**) apical four-chamber view, small atrial defect or patent foramen ovale; (**C**) four-chamber heart view of the xiphoid process, atrial defect or patent foramen ovale.

**Figure 3:**
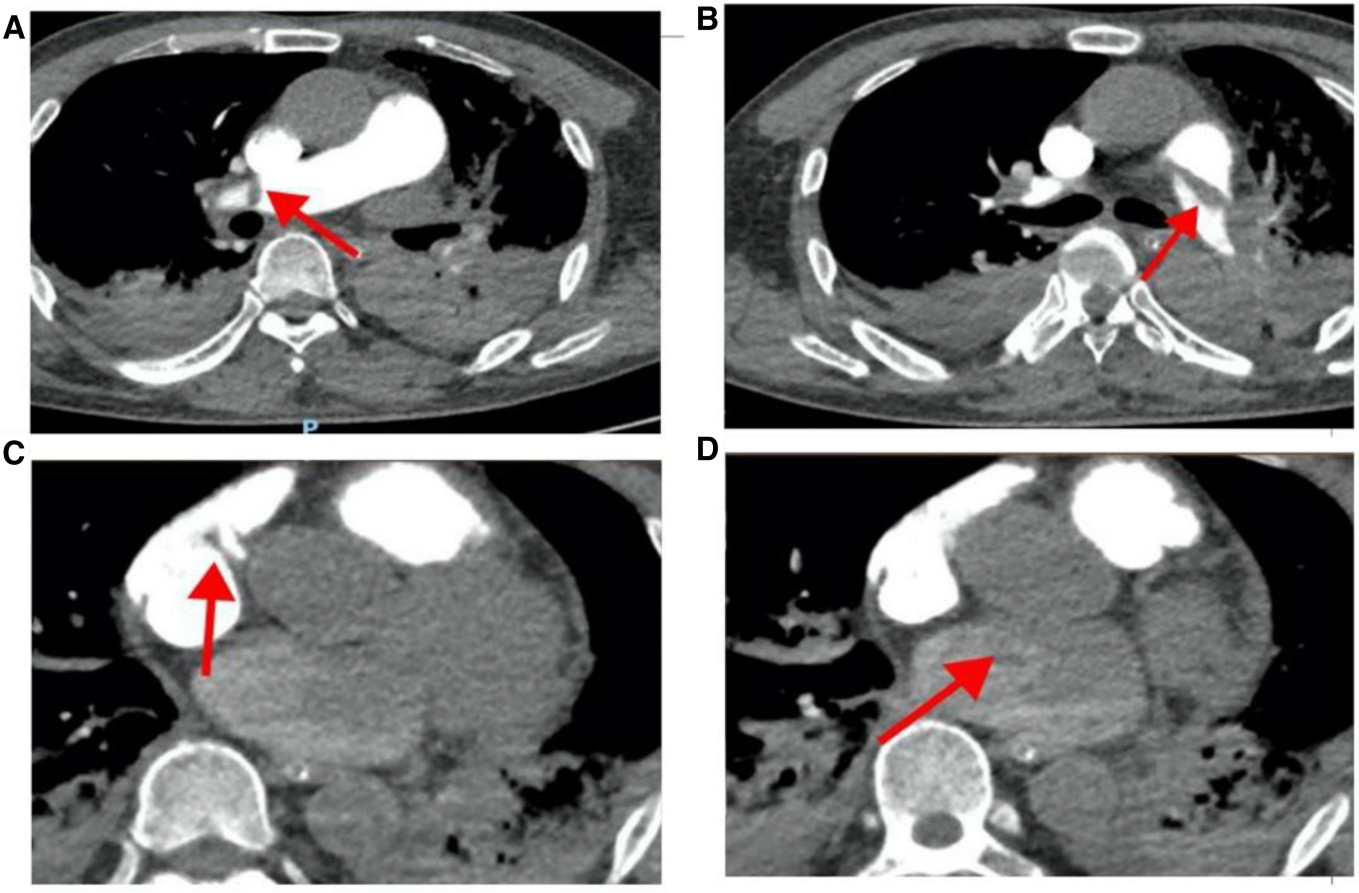
Pulmonary artery CT angiography results when the patient was hospitalized. (**A**) The red arrow points to the filling defect of the right pulmonary artery; (**B**) the red arrow points to the filling defect of the left pulmonary artery; (**C**) the red arrow points to the space in the right atrium; (**D**) the red arrow points to the space in the left atrium.

On March 28, 2023, at 14:20, the patient was transferred to our hospital's Intensive Medicine Department. The patient had a temperature of 36°C, a pulse of 98 beats/min, a respiratory rate of 20 breaths/min, and blood pressure of 92/62 mmHg (under the combined auxiliary treatment with norepinephrine, epinephrine, and dobutamine), Vasoactive Inotropic Score (VIS): 105 points. Pulse oxygen saturation (SpO_2_) was 99%. Blood gas analysis showed a pH of 7.49, blood oxygen concentration of 50%, carbon dioxide partial pressure of 26.00 mmHg, oxygen saturation of 99%, oxygen partial pressure of 106.00 mmHg, blood lactate level of 3.5 mmol/L, and an oxygenation index of 212.00 mmHg. The patient's laboratory test results were Creatinine 176 umol/L, D-dimer >20 mg/L, N-terminal pro-B-type natriuretic peptide (NT-proBNP) 14,535 pg/ml, and cardiac troponin I 1.12 ng/ml. The ECG results indicated a sinus rhythm, abnormal Q waves (Ⅲ, aVF), and T wave changes. Echocardiography showed a left atrial anteroposterior diameter of 26 mm, a right atrial anteroposterior diameter of 46 mm, a left ventricular end-diastolic diameter of 40 mm, a right ventricular anteroposterior diameter of 23 mm, a left ventricular ejection fraction of 54%, thrombi in both atria, severe pulmonary arterial hypertension (70 mmHg), and an atrial septal defect width of approximately 4.1 mm (Figure 2, Table 1). Thrombosis formation was seen in the bilateral popliteal veins (complete occlusion). Pulmonary artery CT angiography indicated bilateral multiple PEs and intra-atrial occupying lesions in both atria (Figure 3).

**Table 1:** The echocardiographic findings and laboratory results before and after the AngioJet procedure.

	Echocardiographic findings						Laboratory results		
	PA pressure	RV size	RA size	LA size	LVEDD	LAEF	NT-Pro-BNP	Troponin	D-dimer
Before AngioJet	70 mmHg	2.3 cm	4.6 cm	2.6 cm	4.0 cm	54%	14,535 pg/ml	1.12 ng/ml	>20 mg/L
After AngioJet	20 mmHg	2.7 cm	3.1 cm	3.4 cm	4.8 cm	17%	11,332 pg/ml	1.59 ng/ml	>20 mg/L
One month later	25 mmHg	2.3 cm	3.2 cm	3.0 cm	5.1 cm	69%	514 pg/ml	0.05 ng/ml	0.88 mg/L

After admission, the patient was initially diagnosed with acute PE (with a high likelihood of thrombi in both atria), and the simplified PE Severity Index score was 2 points. The patient was hemodynamically unstable, with increased troponin and right heart dysfunction, categorizing the risk as high-risk PE. The ECMO expert team at the People's Hospital of Guangxi Zhuang Autonomous Region convened an emergency multidisciplinary consultation involving cardiologists, radiologists, cardiothoracic surgeons, vascular surgeons, and interventional physicians. Considering that the patient could experience exacerbated circulatory failure at any time and the risk of sudden cardiorespiratory arrest, the indications for ECMO were acute circulatory failure caused by PE (12). The recommendation was that the patient undergo further interventional procedure treatment in the interventional department after ECMO assistance.

**Figure 4:**
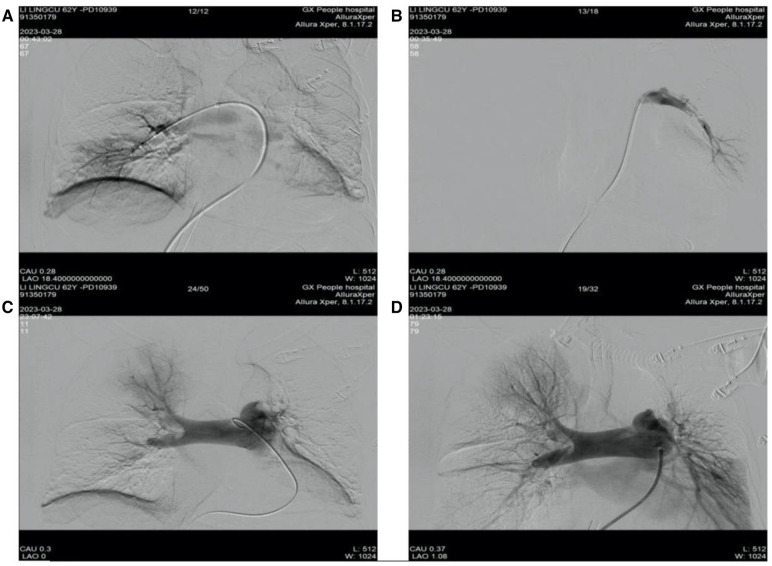
Preoperative and postoperative results of patient's catheterization laboratory interventional surgery. (**A**) Right pulmonary artery thrombus removal; (**B**) left pulmonary artery thrombus removal; (**C**) pulmonary angiography before interventional treatment; (**D**) arrow pulmonary angiography after interventional treatment.

The patient, assisted by ECMO (venoarterial, femoral-femoral), continued anticoagulation therapy with sodium heparin, maintaining an activated partial thromboplastin time (APTT) of 60–80 s. One hour after the switch, the patient was transferred to the interventional catheterization room for pulmonary artery angiography, catheter thrombolysis (using pigtail catheters to fragment the thrombus by continuous rotation of the catheter), and thrombectomy (using the AngioJet system to aspirate the thrombus). During the interventional procedure process, angiography showed a large filling defect in the main trunk of the left pulmonary artery and some branches of the right pulmonary artery, with the left side being more severe, and no opacification in the distal branches of the left pulmonary artery. Therefore, pulmonary artery thrombolysis and thrombectomy were performed. During the thrombectomy, the patient developed ventricular arrhythmia and cardiac arrest, and the heart was restored to spontaneous beating with the administration of adrenaline. Postoperative reexamination with angiography revealed that the main trunks of both the left and right pulmonary arteries had regained patency, the filling defect had markedly improved, and the main branches of both lungs were revisualized, with increased perfusion in both lungs compared to before (Figure 4). The final diagnosis after the patient's interventional procedure was acute PE combined with thrombi in both atria.

**Figure 5:**
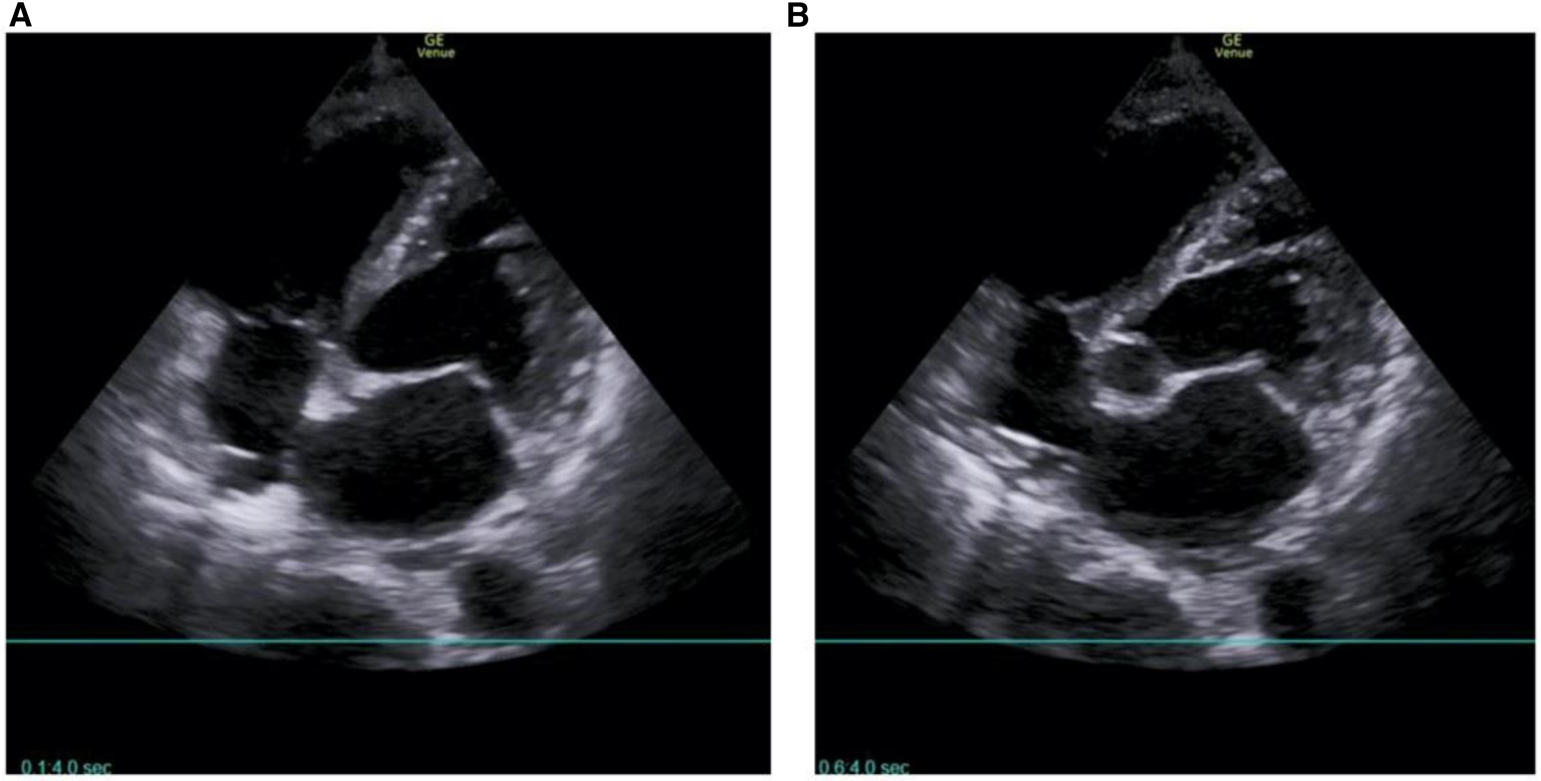
No intracardiac thrombus was found on the echocardiogram of the patient on the first postoperative day. (**A**) Four-chamber heart in the apical region; (**B**) five-chamber heart in the apical region.

On the first day after treatment, the patient's echocardiogram showed a left atrial anteroposterior diameter of 34 mm, a reduced right atrial size compared to before, a left-to-right diameter of 31 mm, a left ventricular end-diastolic diameter of 48 mm, a right ventricular anteroposterior diameter of 27 mm, a left ventricular ejection fraction of 17%, and normal pulmonary artery pressure (20 mmHg) (Figure 5, Table 1). The patient's cardiac contractile function had decreased compared to before, and it was considered that it might have been significantly affected by the cardiac arrest during the interventional procedure. ECMO assistance continued, and continuous anticoagulation with sodium heparin was maintained, keeping APTT between 60 and 80 s.

**Figure 6:**
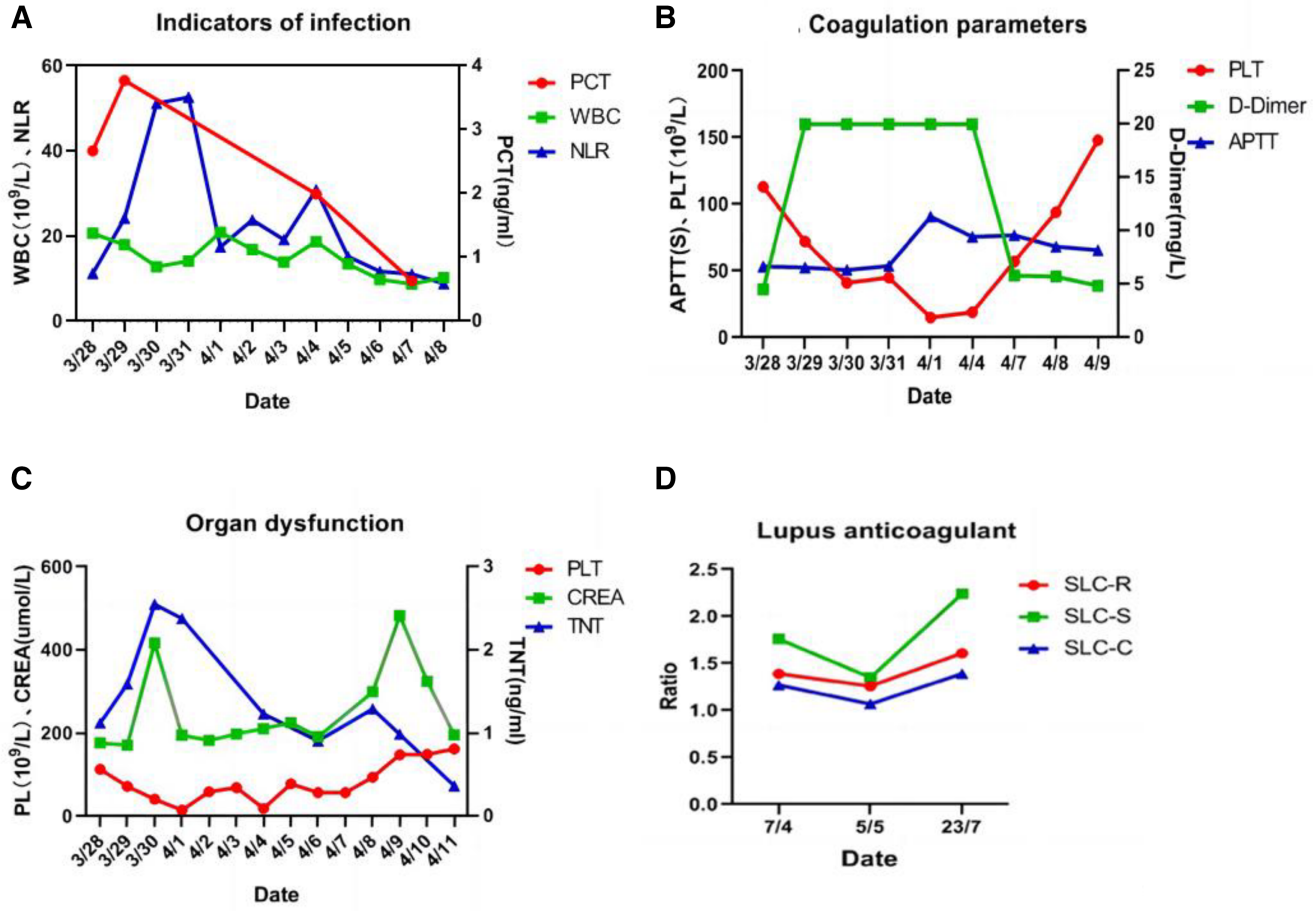
(**A**) Indicators of infection showed a downward trend; (**B**) coagulation parameters showed an improvement trend; (**C**) organ dysfunction showed an improvement trend; (**D**) lupus anticoagulant present in plasma, on ≥2 occasions at least 12 weeks apart.

**Figure 7:**
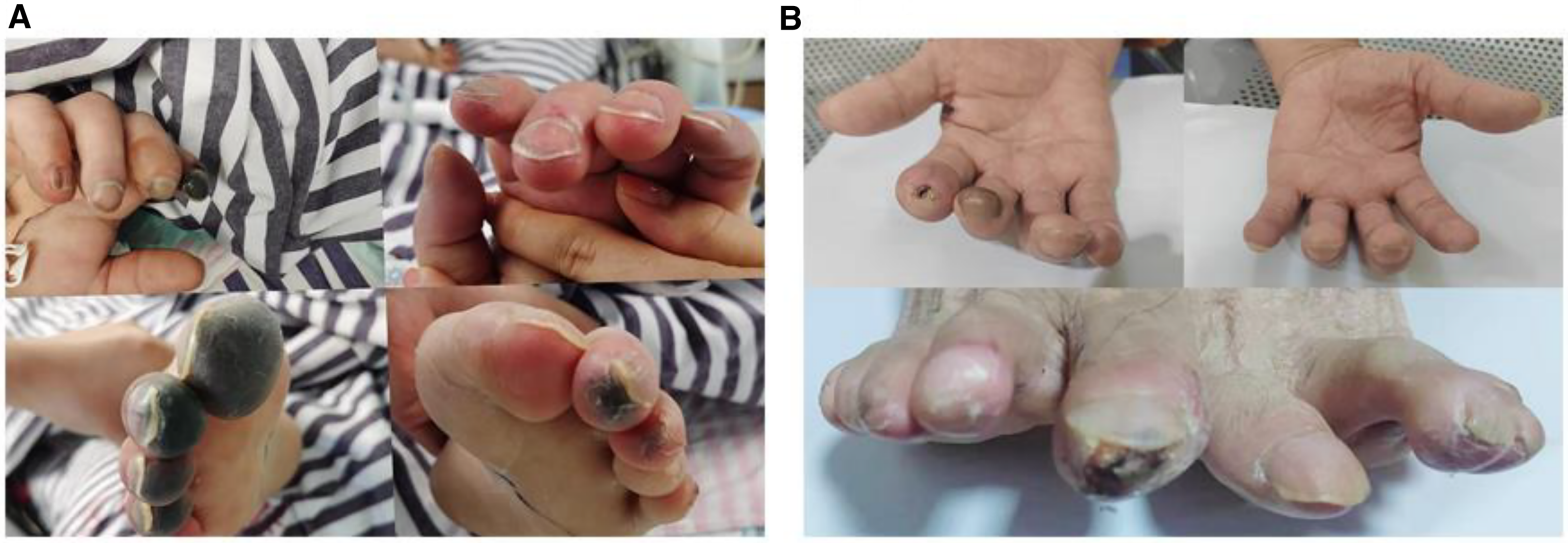
(**A**) Fingers show livedo reticularis and necrosis; (**B**) 6 months later, fingers show no significant livedo reticularis and necrosis.

**Figure 8:**
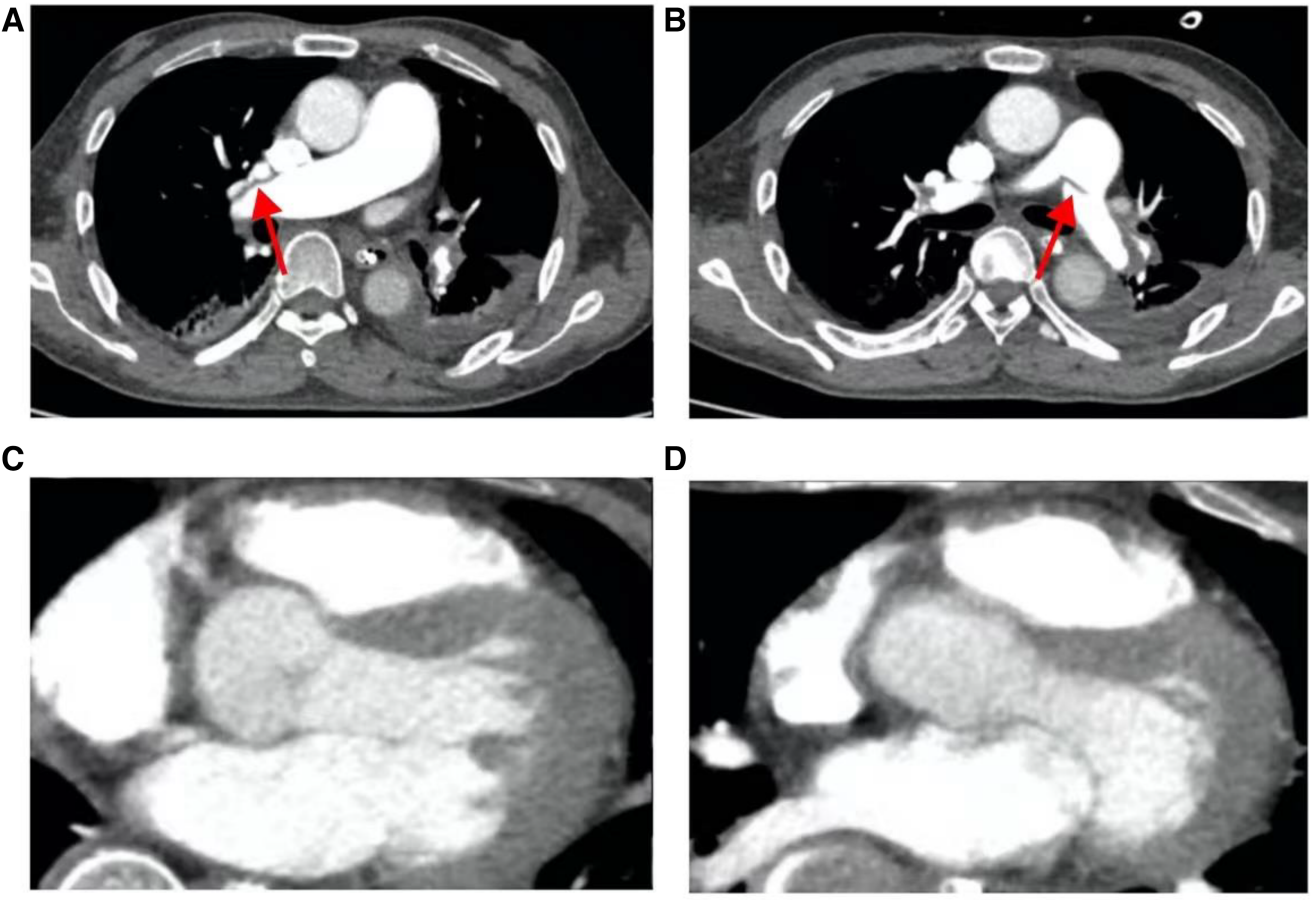
The patient's pulmonary artery CT angiography showed less embolism on the 15th postoperative day than before. (**A**) Slight filling defect in the right pulmonary artery; (**B**) slight filling defect in the left pulmonary artery; (**C**, **D**), the chambers are now mainly patent with maybe minor wall-adherent residuals.

After comprehensive multidisciplinary treatment from the intensive care, cardiology, and interventional departments, the patient's indicators of infection decreased (Figure 6A), cardiac contractile function gradually improved, and his hemodynamics stabilized. Therefore, ECMO was successfully withdrawn on the fifth day post-interventional procedure. The patient's fingers showed livedo reticularis and necrosis (Figure 7A), and enoxaparin was continued. On the tenth day post-interventional procedure, the lupus anticoagulants were again positive, and CAPS was considered after consultation with the rheumatology and immunology departments. The anticoagulation treatment was then adjusted to rivaroxaban, glucocorticoids, and cyclophosphamide were added, multiple organ functions exhibited progressive improvement (Figures 6B,C). The patient was weaned off ventilator support on the 14th day post-interventional procedure. Reexamination of the pulmonary artery angiography on the 15th day indicated that the main pulmonary artery, the left and right pulmonary artery trunks, multiple lobes of both lungs, and segmental arteries showed patchy, cast-like, low-density filling defects, significantly reduced compared to before, and no abnormally thickened or twisted vascular mass was observed (Figure 8). Finally, the patient was discharged on the 20th day after interventional procedure.

A follow-up echocardiogram 1 month after the patient's discharge showed a left atrial anteroposterior diameter of 30 mm, normal right atrial size, a left–right diameter of 32 mm, a left ventricular end-diastolic diameter of 51 mm, a right ventricular anteroposterior diameter of 23 mm, a left ventricular ejection fraction of 69%, and no pulmonary hypertension (Table 1). Pulmonary artery CT angiography showed no filling defects in the main pulmonary arteries or segmental arteries above both lungs. The patient continued to take rivaroxaban and cyclophosphamide for treatment. At the 6-month post-discharge follow-up, the patient reported no discomfort during daily activities, fingers showed no significant livedo reticularis and necrosis (Figure 7B), and laboratory tests, including NT-proBNP and D-dimer, were all within normal ranges.

## Discussion

To our knowledge, this report is the inaugural demonstration of the clinical feasibility and effectiveness of employing AngioJet in conjunction with ECMO for treating a CAPS patient with floating bilateral atrial thrombosis extending to the pulmonary artery and associated with a massive PE. Our findings suggest a novel therapeutic avenue for patients in analogous circumstances or those who are contraindicated for thrombolysis or surgical embolectomy.

Catastrophic Antiphospholipid Syndrome (CAPS) is a critical and potentially fatal autoimmune disease, with an elusive pathogenesis. It is hallmarked by the swift onset of widespread thrombosis within a brief timeframe (usually within a week), culminating in multi-organ failure affecting the kidneys, lungs, heart, and skin. Diagnostic laboratory tests typically reveal the presence of antiphospholipid antibodies (lupus anticoagulants and/or anticardiolipin antibodies). The prevalence of CAPS has been incrementally rising, with common triggers including infections, surgical procedures, or malignancies (13). In 2000, the European Forum for Antiphospholipid Antibodies established the CAPS Registry to systematically document clinical and laboratory data, along with therapeutic interventions for CAPS patients (13). Patients with CAPS often undergo multiple thrombotic episodes, leading to the release of cytokines that induce diffuse vasculopathy syndrome. Patients with CAPS are not easily identified at an early stage, and this can rapidly escalate to ICU admissions due to multi-organ failure. Current therapeutic strategies primarily include anticoagulants, corticosteroids, plasma exchange, and intravenous immunoglobulin therapy, yet the prognosis remains grim with a mortality rate exceeding 30% (2, 14, 15). The first documented case of successful ECMO treatment for refractory cardiogenic shock due to the CAPS was reported in 2010 (16).

The case initially presented with fever and consciousness before quickly developing multi-organ dysfunction. Despite treatment in ICU, the patient's condition necessitated V-A ECMO to address the life-threatening exacerbation of respiratory and circulatory failure. Additionally, the patient was treated using AngioJet thrombectomy for acute large-scale pulmonary embolism associated with bilateral atrial thrombosis. After the patient stabilized, following diagnosis confirmation of CAPS, the patient received specialized treatment including anticoagulants, glucocorticoids, and cyclophosphamide. Eventually, the patient recovered successfully and was discharged from the hospital. AngioJet thrombectomy with V-A ECMO support for an acute large-scale pulmonary embolism with bilateral atrial thrombosis may be the key to successful treatment.

Acute PE combined with biatrial thrombosis is extremely rare. In patients with symptomatic PE, the incidence of intracardiac thrombosis confirmed by echocardiography is only 3.7%, but the mortality rate exceeds 20% (17, 18), and nearly two-thirds of these patients die within 24 h of onset (19). Acute massive PE is also a major cause of pulmonary arterial hypertension and right ventricular failure. For patients with an acute PE with hemodynamic instability, the 30-day mortality rate is 16%–46%; for patients with PE who experience cardiac arrest, the 30-day mortality rate is as high as 52%–84% (5). In this case, the patient developed biatrial thrombosis and pulmonary artery embolism due to the CAPS, leading to increased right heart pressure and opening of the foramen ovale. The patient further developed thrombosis in both atria, leading to a decline in cardiac function and ultimately resulting in hemodynamic instability. Regarding this case, we have two points of experience to summarize. First, for patients with stable respiration and circulation, current guidelines recommend systemic thrombolysis as a first-line treatment strategy to save the lives of high-risk PE patients (17, 20). However, for patients experiencing hemodynamic instability or presenting with deteriorating conditions requiring cardiopulmonary resuscitation due to respiratory or cardiac arrest, the outcome and prognosis of systemic thrombolysis are unfavorable (5). Thus, for such critically ill patients, we can maintain respiratory and circulatory stability through ECMO as a prerequisite and then proceed with pharmacological or mechanical thrombolysis, which may improve the prognosis (21, 22). Second, the treatment strategy for PE combined with biatrial thrombosis is still controversial. However, some centers have reported that interventional treatment or surgical embolectomy can achieve good results (23, 24), and there are case reports indicating that conservative treatment can be adopted (25). Athappan et al. (26) compiled the treatment outcomes of 328 patients with PE combined with simple right heart thrombosis over nearly 20 years, where the mortality rate of patients treated with interventional thrombolysis or surgical treatment was significantly lower than that of patients treated with anticoagulation therapy, but there was no statistically significant difference in survival rates between interventional thrombolysis and surgical treatment. However, some studies also recommend the use of catheter-directed therapy, which involves thrombolysis and embolectomy via interventional catheters. In some PE cases, this has achieved comparatively positive outcomes and prognoses (27–30). According to a consensus statement by the European Society of Cardiology (ESC) Working Group on Pulmonary Circulation and Right Ventricular Function, together with the European Association of Percutaneous Cardiovascular Interventions, there is increasing clinical and scientific interest in catheter-directed therapy (CDT) for managing acute pulmonary embolism (PE). Currently, CDT is recommended for patients with high-risk PE where thrombolysis is contraindicated or has failed. Additionally, CDT is considered a viable option for initially stable patients whose conditions deteriorate despite appropriate anticoagulation therapy (31).

CDT encompasses various methods such as clot fragmentation, mechanical embolectomy, local thrombolysis, and combined pharmaco-mechanical approaches. These techniques primarily function to alleviate thromboembolic blockages in the proximal pulmonary arteries, thereby restoring pulmonary blood flow and enhancing right ventricular (RV) function. Local thrombolysis can be administered using pigtail or specialized sidehole catheters. Despite the availability of multiple CDT techniques, there is no conclusive evidence favoring one over the others, leading to significant variability in periprocedural anticoagulation practices. It is important to note that complete thrombus removal from the pulmonary vasculature is typically unnecessary for achieving hemodynamic stability. Indicators such as increased systemic pressure, improved blood gas parameters, and reduced heart rate signify a reduction in pulmonary obstruction and enhanced RV function, which can often be attained through partial recanalization (31). AngioJet rheolytic thrombectomy (ART) is extensively used in clinical practice. It was initially utilized in deep vein thrombosis diseases, can quickly remove a thrombosis and restore venous patency with a relatively straightforward procedure and high therapeutic efficiency, and without causing significant damage to the venous wall and valve (32). However, there are limited studies on the application of AngioJet in PE, and many reports involve a small number of cases, which may be related to the disease characteristics of severe PE. A study conducted showed that technical success was obtained in 92.2% of the patients, with a significant improvement in obstruction, perfusion, and Miller indexes in each subgroup (33). A feasibility pilot study on AngioJet rheolytic thrombectomy in patients presenting with high-risk PE and cardiogenic shock did not find significant improvement in the prognosis of high-risk PE (34). A study reported two cases of submassive PE accompanied by right ventricular dysfunction and pulmonary hypertension. Both patients were successfully treated with the AngioJet system (35). Some research shows that the management of high- and intermediate-risk PE with percutaneous mechanical pulmonary thrombectomy (PMPT) is technically feasible, relatively safe, and effective (36).

The use of AngioJet has been linked to complications such as bradycardia, blockage, and even procedural mortality, leading the FDA to recommend against its use as a first-line treatment for PE (37). However, a recent systematic review and meta-analysis reported that the overall pooled rates of bleeding events, bradycardia, worsening renal function episodes, PE-related mortality, and all-cause mortality suggest favorable outcomes for the efficacy and safety of ART in specific acute PE scenarios. This suggests that a reassessment of the FDA's black-box warning on ART may be warranted (38). In this case, the patient underwent an AngioJet procedure for interventional thrombectomy with the support of ECMO (39). Although ventricular arrhythmia and cardiac arrest occurred during the procedure, the patient remained stable with the support of ECMO, so the operation was completed safely, and his condition improved rapidly. Interventional treatment is minimally invasive, a low bleeding risk, and a good thrombus removal effect. It is suitable for patients who cannot tolerate surgery or who do not meet the conditions for surgery.

When encountering patients with acute PE, the presence of an intracardiac thrombus should be actively investigated, and the severity of acute PE should be assessed concurrently. Some research results show that when patients with PE develop hemodynamic instability and could worsen at any time and suffer cardiac arrest, using VA-ECMO to assist with embolectomy or thrombolysis results in a better survival rate than thrombolysis alone (39). Patients with acute PE combined with biatrial thrombosis are rare and have a high mortality rate, and active treatment measures should be taken in clinical practice.

## Conclusions

Catastrophic Antiphospholipid Syndrome (CAPS) is a critical condition characterized by extensive multi-organ thrombosis and functional impairment. In cases where patients present with acute pulmonary embolism (PE) and intracardiac, particularly biatrial, space-occupying lesions, the rapid assembly of a multidisciplinary emergency team is imperative for determining an appropriate treatment strategy. Such clinical scenarios are prone to precipitate severe respiratory and circulatory failure, significantly influencing patient outcomes. Prompt initiation of Extracorporeal Membrane Oxygenation (ECMO) therapy offers a vital window for effectively addressing the root cause. While ECMO support enhances the feasibility of interventional or surgical thrombectomy, a thorough risk assessment encompassing potential bleeding, trauma severity, and the patient's surgical tolerance is essential. Interventional approaches may present distinct advantages, the AngioJet procedure device presents as a viable treatment alternative. However, in cases complicated by malignancies or immune disorders, a multidisciplinary dialogue is crucial to develop an optimal treatment plan.

## Data Availability

The original contributions presented in the study are included in the article/Supplementary Material, further inquiries can be directed to the corresponding author.
